# Synthesis and structure of 2,4,6-tri­cyclo­butyl-1,3,5-trioxane

**DOI:** 10.1107/S205698901900896X

**Published:** 2019-10-03

**Authors:** Sergey V. Shorunov, Maxim V. Bermeshev, Dmitry V. Demchuk, Yulia V. Nelyubina

**Affiliations:** a A.V.Topchiev Institute of Petrochemical Synthesis, Russian Academy of Sciences, 29 Leninsky prospect, 119991, Moscow, Russian Federation; b N.D. Zelinsky Institute of Organic Chemistry, Russian Academy of Sciences, 47, Leninsky prospect, 119991, Moscow, Russian Federation; c A.N. Nesmeyanov Institute of Organoelement Compounds, Russian Academy of, Sciences, 28 Vavilova Str, Moscow, 119991, Russian Federation

**Keywords:** trioxane, cyclo­butane, cyclo­butane carbaldehyde, strained rings, swern oxidation, crystal structure

## Abstract

The synthesis and structure of 2,4,6,-tri­cyclo­butyl-1,3,5-trioxane, C_15_H_24_O_3_ **1**, is described. It was formed in 39% yield during the work-up of the Swern oxidation of cyclo­butyl­methanol and may serve as a stable precursor of the cyclo­butane carbaldehyde. The mol­ecule of **1** occupies a special position (3.*m*) located at the center of its 1,3,5-trioxane ring. The latter is in a chair conformation, with the symmetry-independent O and C atoms deviating by 0.651 (4) Å from the least-squares plane of the other atoms of the trioxane ring. All three cyclo­butane substituents, which have a butterfly conformation with an angle between the two planes of 25.7 (3)°, are in the *cis* conformation relative to the 1,3,5-trioxane ring. Inter­molecular C—H⋯O inter­actions between the 1,3,5-trioxane rings consolidate the crystal structure, forming stacks along the *c*-axis direction. The crystal studied was refined a as a racemic twin.

## Abstract 2

The synthesis and structure of the substituted trioxane compound 2,4,6,-tri­cyclo­butyl-1,3,5-trioxane is described. The three cyclo­butane rings attached to the 1,3,5-trioxane six-membered ring are all in a *cis* arrangement. The compound may find application as a stable trimeric form of the fragile cyclo­butane carbaldehyde.

## Chemical context   

Aliphatic aldehydes are well known to form trimers – derivatives of 1,3,5-trioxane. Some of these trimers are commercially-available, stable forms of the corresponding aldehydes, from which monomers can be regenerated by simple heating. Examples are trioxane and paraldehyde. For our research in the area of polymer chemistry, we need regular amounts of cyclo­butane carbaldehyde. Besides this, cyclo­butane carbaldehyde is a versatile synthetic building block for the incorporation of the cyclo­butane ring into organic mol­ecules in medicinal chemistry and drug discovery (Deaton *et al.*, 2008 ▸; Inagaki *et al.*, 2011 ▸). However, it is known to be unstable and prone to polymerization and decomposition by various routes (Slobodin & Blinova, 1953 ▸; Roquitte & Walters, 1962 ▸; Funke & Cerfontain, 1976 ▸). Bearing all that in mind, we assumed that the trimeric form of cyclo­butane carbaldehyde should have a long shelf life and might find application as a stable equivalent of cyclo­butane carbaldehyde for long-term storage, with the possibility of performing a thermal breakdown back to the monomer once required. In this paper we describe our attempted synthesis of 2,4,6,-tri­cyclo­butyl-1,3,5-trioxane, **1**, the trimer of cyclo­butane carbaldehyde.

**Figure 1:**
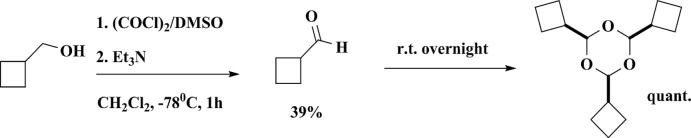
Synthesis of 2,4,6-tri­cyclo­butyl-1,3,5-trioxane, **1**.

One of the most straightforward routes to cyclo­butane carbaldehyde is the oxidation of the commercially available cyclo­butyl­methanol (Yusubov *et al.*, 2007 ▸; Deaton *et al.* 2008 ▸). Further treatment with calcium chloride (Slobodin & Blinova, 1953 ▸) should afford the desired trimer. By employing Swern oxidation conditions, we were able to obtain the desired aldehyde monomer in 39% yield. (Fig. 1 ▸). It was found that freshly distilled cyclo­butane carbaldehyde solidifies quickly upon short storage at room temperature, forming a colourless solid material readily soluble in organic solvents, especially non-polar ones. No calcium chloride was required for the trimerization, contrary to what was observed by Slobodin and co-workers. It should be noted that the melting point of our trimer was 391–393 K, close to that reported by Slobodin (391.5–393.5 K) while Funke and co-workers observed the melting to proceed at 397–398 K (Funke & Cerfontain, 1976 ▸). Analysis of the synthesized compound by means of ^1^H and ^13^C NMR spectroscopy revealed the presence of unchanged cyclo­butane rings along with the absence of the aldehyde group [no downfield signals of the aldehyde proton (*ca* 9–12 ppm) in the ^1^H NMR spectrum and no signals of the aldehyde carbon (*ca* 180-220 ppm) in the ^13^C NMR spectrum]. The most downfield signal in the ^1^H NMR spectrum was a doublet at 4.81 ppm and the most downfield signal in the ^13^C NMR spectrum was observed at 103.3 ppm. The number of signals and their integral intensities (in the ^1^H NMR spectrum) demonstrated that, aside from the disappearance of the aldehyde group, no further degradation of the parent mol­ecule had taken place. We made an assumption that the desired trimer had formed. However, dimeric, trimeric, tetra­meric and higher cyclic forms or a linear polymer could also form. It should be noticed that the NMR technique cannot distinguish between these forms, since all the integral intensities and coupling constants would be the same, unless a large macrocycle is formed, where conformational effects would make some equivalent signals become non-equivalent. The mass spectrum of **1** solved the question and proved the structure to be trimeric. However, neither NMR nor mass spectrometry can provide information about the assignment of the cyclo­butyl groups with respect to the trioxane ring. It is known that paraldehyde – the trimer of acetaldehyde – exists in the form of two structural isomers: the *cis* isomer with all three methyl groups being in equatorial positions with respect to the trioxane ring and the *trans* isomer with one methyl group in an axial position and the two methyl groups in equatorial positions (Carpenter & Brockway,1936 ▸; Kewley, 1970 ▸). These structures have the smallest 1,3-diaxial strain and are the only ones observed. In our case, with more bulky cyclo­butyl groups, one might expect that only the *cis* isomer would be formed.

The mol­ecular structure of 2,4,6-tri­cyclo­butyl-1,3,5-trioxane, **1**, has been elucidated by X-ray diffraction analysis on single crystals grown from methanol. In summary, 2,4,6,-tri­cyclo­butyl-1,3,5-trioxane, **1**, was formed in 39% yield during the work-up of the oxidation reaction of cyclo­butyl­methanol with DMSO and oxalyl chloride (Swern oxidation). It is a trimeric form of the cyclo­butane carbaldehyde. The aldehyde itself is unstable and quickly polymerizes even at room temperature. The described trimer could probably serve as a stable form of the cyclo­butane carbaldehyde. The compound is also of potential inter­est as a solid fuel.
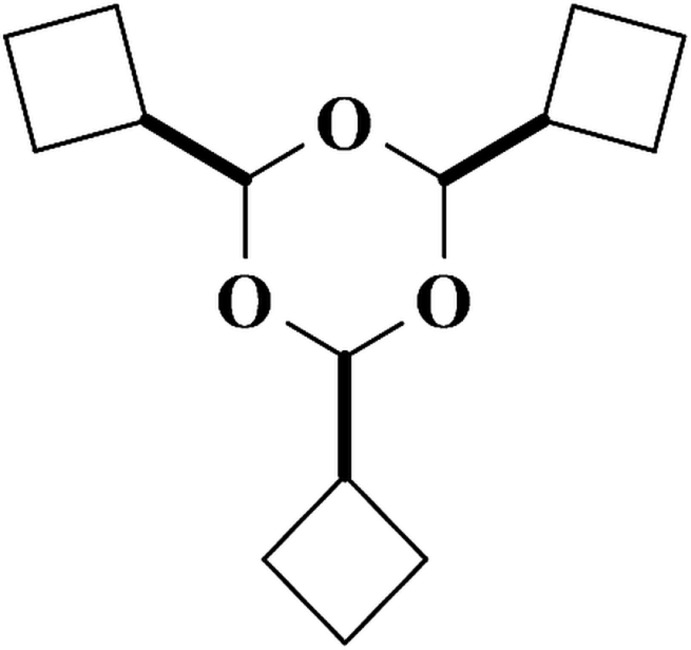


## Structural commentary   

**Figure 2:**
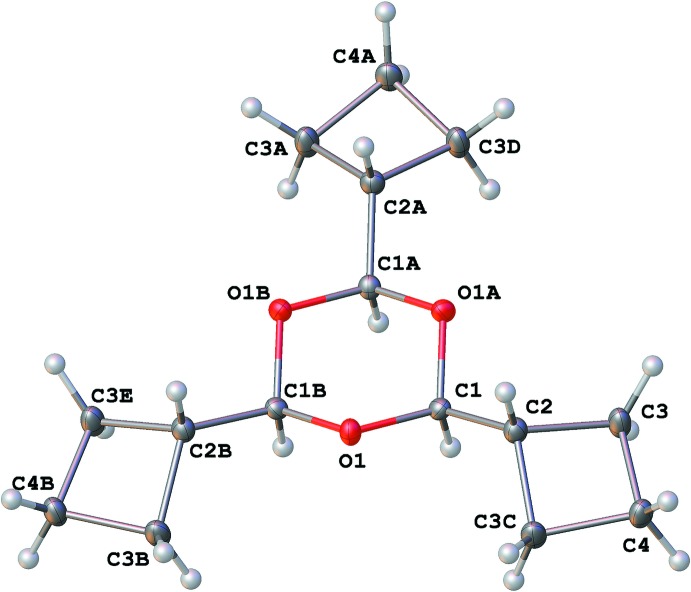
Mol­ecular structure of **1** with displacement ellipsoids drawn at the 50% probability level. Symmetry codes: (A) *y* − *x*, −*x*, *z;* (B) −*y*, *x* − *y*, *z*; (C) *y*, *x*, *z*; (D) −*y* + *x*, −*y*, *z*; (E) −*x*, −*x* + *y*, *z*.

The mol­ecule of **1** (Fig. 2 ▸) occupies a special position (3.*m*) occurring in the center of its 1,3,5-trioxane ring. The latter is in a chair conformation with the symmetry-independent atoms O1 and C1 deviating by 0.651 (4) Å from the least-squares plane of the other ring atoms. All three cyclo­butane substituents, which show a butterfly conformation with an angle between the two planes of 25.7 (3)°, are in a *cis* conformation relative to the 1,3,5-trioxane ring.

## Supra­molecular features   

**Figure 3:**
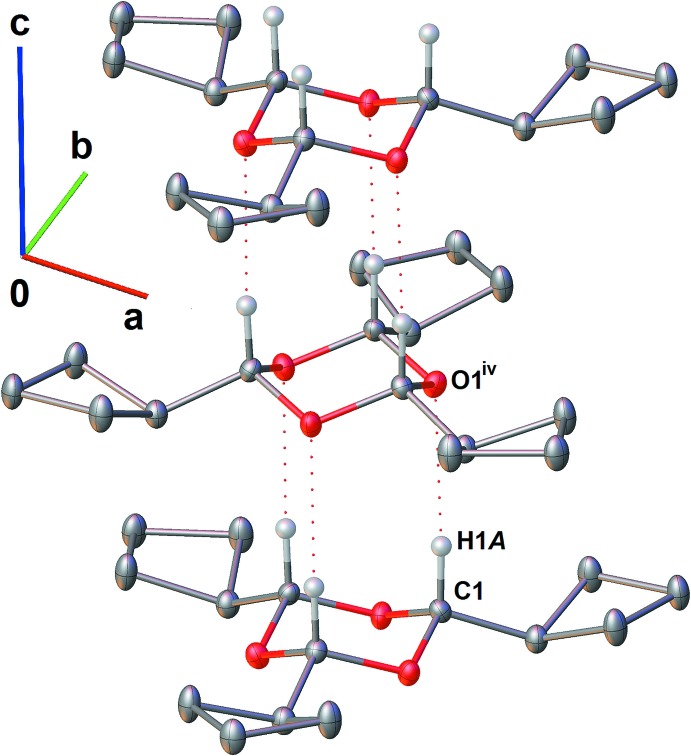
A fragment of the infinite stacks formed by mol­ecules of **1** along the *c*-axis direction. Hydrogen atoms except those of the 1,3,5-trioxane rings are omitted for clarity. Red dashed lines represent inter­molecular C—H⋯O inter­actions (Table 1 ▸).

Infinite stacks of mol­ecules of **1** along the *c-*axis direction consolidate the crystal structure (Fig. 3 ▸) through C1—H1*A*⋯O1^iv^ inter­actions between adjacent 1,3,5-trioxane rings (Table 1 ▸).

## Database survey   

The *cis*–*cis*–*cis* configuration of the cyclo­butane rings observed in **1** seems to be typical for 2,4,6-tris­ubstituted-1,3,5-trioxanes, as follows from the search for such compounds in the Cambridge Structural Database (CSD, Version 5.40, November 2018 update; Groom *et al*, 2016 ▸). Indeed, only in 2,4,6-tris­(tri­chloro­meth­yl)-1,3,5-trioxane (refcode PRCHLA; Hay & Mackay, 1980 ▸) out of the 31 entries found, is one of the three substituents in a *trans* conformation with the other two are in a *cis* conformation. Another compound (MUKCEC; Arias-Ugarte *et al.*, 2015 ▸) consists of as a superposition of both *cis*–*cis*–*cis* and *trans*–*cis*–*cis* conformations as a result of the disorder of one of the subsitutents. In the latter conformation, the central ring is in a boat conformation as the equatorially oriented cyclobutane rings become axially oriented.

## Synthesis and crystallization   

**General experimental remarks**

The synthesis of **1** was carried out under a purified argon atmosphere. The ^1^H and ^13^C NMR spectra were recorded on a Varian MercuryPlus 300 (300 MHz) spectrometer using CDCl_3_ as solvent. Di­chloro­methane, tri­ethyl­amine and DMSO were distilled over calcium hydride. Methanol was distilled over magnesium turnings. Oxalyl chloride was distilled over phospho­rus pentoxide. Cyclo­butyl­methanol is commercially available and was used without further purification.

**Synthesis of compound 1**

To a stirred solution of oxalyl chloride (13.5 g, 0.11 mol) in 110 ml of dry di­chloro­methane was added dropwise at 195K the solution of dry di­methyl­sulfoxide (16.5 g, 0.21 mol) in 30 ml of dry di­chloro­methane followed by the solution of cyclo­butyl­methanol (8.1 g, 0.09 mol) in 110 ml of dry di­chloro­methane. The reaction mixture was stirred at 195 K for 1 h and then neat tri­ethyl­amine (48.2 g, 0.47 mol) was added. The cooling bath was removed and when the reaction mixture had returned to room temperature, 100 ml of water was added and the reaction mixture was further stirred for 10 min. The flask content was then transferred to a separatory funnel, the bottom organic phase was collected and the aqueous phase was extracted with di­chloro­methane (2 × 100 ml). The combined organic phase was washed with 10% HCl (3 × 100 ml), water (2 × 100 ml), dried with anhydrous magnesium sulfate, filtered and the solvent was removed on the rotary evaporator. The residue was distilled under atmospheric pressure and the fraction boiling in the range 399–393 K was collected. The yield was 3.09 g (39%) and the product solidified after standing at room temperature overnight, qu­anti­tatively forming compound **1**. ^1^H NMR (300 MHz, CDCl_3_): δ 1.65–2.16 (6H, *m*), 2.55 (1H, *dt*, *J* = 15.7; 7.7 Hz), 4.81 (1H, *d*, *J* = 5.6 Hz). ^13^C NMR (75 MHz, CDCl_3_): δ 18.5; 22.4; 38.1; 103.3. Mass spectrum *m*/*z*: 252 ([*M* ^+^]), 169, 85, 67.

Colourless crystals of **1** suitable for X-ray analysis were obtained by dissolving the crude solid material (0.05 g) in 5 ml of warm methanol and keeping the resulting solution in a vial tightly stoppered with a plug of cotton wool for two days at room temperature.

## Refinement   

Crystal data, data collection and structure refinement details are summarized in Table 2 ▸. The hydrogen atoms were positioned geometrically (C—H = 1.00 and 0.99 Å for CH and CH_2_ groups, respectively) and refined using a riding model with *U*_iso_(H) = 1.2*U*_eq_(C). The crystal studied was refined as a racemic twin with a BASF of 0.146.

## Supplementary Material

Crystal structure: contains datablock(s) Trioxane. DOI: 10.1107/S205698901900896X/kq2025sup1.cif

CCDC reference: 1854532

Additional supporting information:  crystallographic information; 3D view; checkCIF report

## Figures and Tables

**Table 1:** Hydrogen-bond geometry (Å, °)

*D*—H⋯*A*	*D*—H	H⋯*A*	*D*⋯*A*	*D*—H⋯*A*
C1—H1*A*⋯O1^iv^	1.00	2.52	3.514 (3)	177

Symmetry code: (iv) 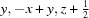.

**Table 2:** Experimental details

Crystal data	
Chemical formula	C_15_H_24_O_3_
*M*_r_	252.34
Crystal system, space group	Hexagonal, *P*6_3_ *c* *m*
Temperature (K)	120
*a*, *c* (Å)	9.9966 (12), 7.9461 (10)
*V* (Å^3^)	687.68 (19)
*Z*	2
Radiation type	Mo *K*α
μ (mm^−1^)	0.08
Crystal size (mm)	0.30 × 0.25 × 0.20

Data collection	
Diffractometer	Bruker APEXII DUO CCD area detector
Absorption correction	Multi-scan (*SADABS*; Bruker, 2008 ▸)
*T*_min_, *T*_max_	0.701, 0.746
No. of measured, independent and observed [*I* > 2σ(*I*)] reflections	7859, 671, 645
*R*_int_	0.026
(sin θ/λ)_max_ (Å^−1^)	0.681

Refinement	
*R*[*F* ^2^ > 2σ(*F* ^2^)], *wR*(*F* ^2^), *S*	0.041, 0.108, 1.03
No. of reflections	671
No. of parameters	35
No. of restraints	1
H-atom treatment	H-atom parameters constrained
Δρ_max_, Δρ_min_ (e Å^−3^)	0.36, −0.22
Absolute structure	Refined as an inversion twin

Computer programs: *APEX2* and *SAINT* (Bruker, 2008 ▸), *SHELXT* (Sheldrick, 2015*a* ▸) and *SHELXL2014/6* (Sheldrick, 2015*b* ▸).

## supplementary crystallographic information

### Crystal data

**Table:**

C_15_H_24_O_3_	*D*_x_ = 1.219 Mg m^−^^3^
*M**_r_* = 252.34	Mo *K*α radiation, λ = 0.71073 Å
Hexagonal, *P*6_3_*c**m*	Cell parameters from 7859 reflections
*a* = 9.9966 (12) Å	θ = 3–30°
*c* = 7.9461 (10) Å	µ = 0.08 mm^−^^1^
*V* = 687.68 (19) Å^3^	*T* = 120 K
*Z* = 2	Prism, colorless
*F*(000) = 276	0.30 × 0.25 × 0.20 mm

### Data collection

**Table:**

Bruker APEXII DUO CCD area detector diffractometer	645 reflections with *I* > 2σ(*I*)
phi and ω scans	*R*_int_ = 0.026
Absorption correction: multi-scan (*SADABS*; Bruker, 2008)	θ_max_ = 29.0°, θ_min_ = 2.4°
*T*_min_ = 0.701, *T*_max_ = 0.746	*h* = −13→13
7859 measured reflections	*k* = −13→13
671 independent reflections	*l* = −10→10

### Refinement

**Table:**

Refinement on *F*^2^	Hydrogen site location: inferred from neighbouring sites
Least-squares matrix: full	H-atom parameters constrained
*R*[*F*^2^ > 2σ(*F*^2^)] = 0.041	*w* = 1/[σ^2^(*F*_o_^2^) + (0.0819*P*)^2^ + 0.1148*P*] where *P* = (*F*_o_^2^ + 2*F*_c_^2^)/3
*wR*(*F*^2^) = 0.108	(Δ/σ)_max_ < 0.001
*S* = 1.03	Δρ_max_ = 0.36 e Å^−^^3^
671 reflections	Δρ_min_ = −0.22 e Å^−^^3^
35 parameters	Absolute structure: Refined as an inversion twin
1 restraint	

### Special details

**Table:**

Geometry. All esds (except the esd in the dihedral angle between two l.s. planes) are estimated using the full covariance matrix. The cell esds are taken into account individually in the estimation of esds in distances, angles and torsion angles; correlations between esds in cell parameters are only used when they are defined by crystal symmetry. An approximate (isotropic) treatment of cell esds is used for estimating esds involving l.s. planes.

### Fractional atomic coordinates and isotropic or equivalent isotropic displacement parameters (Å^2^)

**Table:**

	*x*	*y*	*z*	*U*_iso_*/*U*_eq_	
O1	0.0000	0.13467 (13)	0.4774 (2)	0.0133 (4)	
C1	0.13482 (17)	0.13482 (17)	0.5351 (3)	0.0120 (5)	
H1A	0.1384	0.1384	0.6609	0.014*	
C2	0.2745 (2)	0.2745 (2)	0.4645 (3)	0.0149 (4)	
H2A	0.2716	0.2716	0.3387	0.018*	
C3	0.43522 (17)	0.31060 (18)	0.5281 (3)	0.0198 (4)	
H3A	0.4938	0.2852	0.4462	0.024*	
H3B	0.4334	0.2667	0.6403	0.024*	
C4	0.4839 (2)	0.4839 (2)	0.5319 (4)	0.0192 (5)	
H4A	0.5420	0.5420	0.4310	0.023*	
H4B	0.5377	0.5377	0.6365	0.023*	

### Atomic displacement parameters (Å^2^)

**Table:**

	*U*^11^	*U*^22^	*U*^33^	*U*^12^	*U*^13^	*U*^23^
O1	0.0101 (7)	0.0122 (6)	0.0170 (7)	0.0050 (3)	0.000	0.0017 (5)
C1	0.0113 (7)	0.0113 (7)	0.0135 (9)	0.0057 (6)	−0.0016 (7)	−0.0016 (7)
C2	0.0123 (7)	0.0123 (7)	0.0193 (8)	0.0056 (7)	0.0002 (7)	0.0002 (7)
C3	0.0118 (6)	0.0142 (7)	0.0339 (8)	0.0068 (6)	−0.0022 (7)	−0.0008 (8)
C4	0.0124 (7)	0.0124 (7)	0.0304 (9)	0.0044 (7)	−0.0014 (10)	−0.0014 (10)

### Geometric parameters (Å, º)

**Table:**

O1—C1	1.4229 (13)	C2—H2A	1.0000
O1—C1^i^	1.4230 (13)	C3—C4	1.548 (2)
C1—O1^ii^	1.4229 (13)	C3—H3A	0.9900
C1—C2	1.505 (3)	C3—H3B	0.9900
C1—H1A	1.0000	C4—C3^iii^	1.548 (2)
C2—C3	1.545 (2)	C4—H4A	0.9900
C2—C3^iii^	1.545 (2)	C4—H4B	0.9900
			
C1—O1—C1^i^	110.23 (18)	C2—C3—C4	88.62 (11)
O1^ii^—C1—O1	110.03 (17)	C2—C3—H3A	113.9
O1^ii^—C1—C2	108.66 (11)	C4—C3—H3A	113.9
O1—C1—C2	108.66 (11)	C2—C3—H3B	113.9
O1^ii^—C1—H1A	109.8	C4—C3—H3B	113.9
O1—C1—H1A	109.8	H3A—C3—H3B	111.1
C2—C1—H1A	109.8	C3—C4—C3^iii^	88.39 (15)
C1—C2—C3	117.97 (13)	C3—C4—H4A	113.9
C1—C2—C3^iii^	117.97 (13)	C3^iii^—C4—H4A	113.9
C3—C2—C3^iii^	88.59 (16)	C3—C4—H4B	113.9
C1—C2—H2A	110.2	C3^iii^—C4—H4B	113.9
C3—C2—H2A	110.2	H4A—C4—H4B	111.1
C3^iii^—C2—H2A	110.2		
			
C1^i^—O1—C1—O1^ii^	58.4 (3)	O1—C1—C2—C3^iii^	67.9 (2)
C1^i^—O1—C1—C2	177.20 (10)	C1—C2—C3—C4	−139.29 (18)
O1^ii^—C1—C2—C3	−67.9 (2)	C3^iii^—C2—C3—C4	−18.09 (19)
O1—C1—C2—C3	172.39 (15)	C2—C3—C4—C3^iii^	18.05 (19)
O1^ii^—C1—C2—C3^iii^	−172.39 (15)		

Symmetry codes: (i) −*y*, *x*−*y*, *z*; (ii) −*x*+*y*, −*x*, *z*; (iii) *y*, *x*, *z*.

### Hydrogen-bond geometry (Å, º)

**Table:**

*D*—H···*A*	*D*—H	H···*A*	*D*···*A*	*D*—H···*A*
C1—H1*A*···O1^iv^	1.00	2.52	3.514 (3)	177

Symmetry code: (iv) *y*, −*x*+*y*, *z*+1/2.
